# Corticotropin-Releasing Hormone Receptor Type 1 (CRHR1) Clustering with MAGUKs Is Mediated via Its C-Terminal PDZ Binding Motif

**DOI:** 10.1371/journal.pone.0136768

**Published:** 2015-09-09

**Authors:** Julia Bender, Maik Engeholm, Marion S. Ederer, Johannes Breu, Thor C. Møller, Stylianos Michalakis, Tamas Rasko, Erich E. Wanker, Martin Biel, Karen L. Martinez, Wolfgang Wurst, Jan M. Deussing

**Affiliations:** 1 Max Planck Institute of Psychiatry, Department of Stress Neurobiology and Neurogenetics, Molecular Neurogenetics, Munich, Germany; 2 Max Planck Institute of Psychiatry, Munich, Germany; 3 University of Copenhagen, Department of Chemistry & Nano-Science Center, Copenhagen, Denmark; 4 Center for Integrated Protein Science Munich (CIPSM) and Department of Pharmacy-Center for Drug Research, Ludwig-Maximilians-Universität München, Munich, Germany; 5 Max Delbrueck Center for Molecular Medicine, Berlin-Buch, Germany; 6 Institute of Developmental Genetics, Helmholtz Center Munich, German Research Center for Environmental Health, Neuherberg, Germany; 7 German Center for Neurodegenerative Diseases within the Helmholtz Association, Munich, Germany; 8 Technische Universität München-Weihenstephan, Lehrstuhl für Entwicklungsgenetik c/o Helmholtz Zentrum München, Neuherberg, Germany; University of Toledo, UNITED STATES

## Abstract

The corticotropin-releasing hormone receptor type 1 (CRHR1) plays an important role in orchestrating neuroendocrine, behavioral, and autonomic responses to stress. To identify molecules capable of directly modulating CRHR1 signaling, we performed a yeast-two-hybrid screen using the C-terminal intracellular tail of the receptor as bait. We identified several members of the membrane-associated guanylate kinase (MAGUK) family: postsynaptic density protein 95 (PSD95), synapse-associated protein 97 (SAP97), SAP102 and membrane associated guanylate kinase, WW and PDZ domain containing 2 (MAGI2). CRHR1 is co-expressed with the identified MAGUKs and with the additionally investigated PSD93 in neurons of the adult mouse brain and in primary hippocampal neurons, supporting the probability of a physiological interaction *in vivo*. The C-terminal PDZ (PSD-95, discs large, zona occludens 1) binding motif of CRHR1 is essential for its physical interaction with MAGUKs, as revealed by the CRHR1-STAVA mutant, which harbors a functionally impaired PDZ binding motif. The imitation of a phosphorylation at Thr413 within the PDZ binding motif also disrupted the interaction with MAGUKs. In contrast, distinct PDZ domains within the identified MAGUKs are involved in the interactions. Expression of CRHR1 in primary neurons demonstrated its localization throughout the neuronal plasma membrane, including the excitatory post synapse, where the receptor co-localized with PSD95 and SAP97. The co-expression of CRHR1 and respective interacting MAGUKs in HEK293 cells resulted in a clustered subcellular co-localization which required an intact PDZ binding motif. In conclusion, our study characterized the PDZ binding motif-mediated interaction of CRHR1 with multiple MAGUKs, which directly affects receptor function.

## Introduction

The corticotropin-releasing hormone receptor type 1 (CRHR1) is an important regulator of neuroendocrine, behavioral, and autonomic response to stress [1]. Dysregulation of CRHR1 and its ligand CRH has been causally linked to stress-related pathologies including mood and anxiety disorders. Increased levels of CRH in the cerebrospinal fluid and reduced CRH binding sites in the frontal cortex—probably secondary to elevated CRH concentration—have been reported in depressed subjects [2, 3]. The conditional inactivation of CRHR1 within the murine forebrain demonstrated that limbic CRHR1 signaling modulates anxiety-related behavior, independent of its role in the neuroendocrine stress response via the hypothalamic–pituitary–adrenocortical axis [4]. We have recently identified a bidirectional control of anxiety-related behavior by CRHR1 in anxiogenic glutamatergic and anxiolytic dopaminergic circuits [5], suggesting distinct CRHR1-dependent signaling pathways. CRHR1 is a G protein-coupled receptor (GPCR) of the B1 family and preferentially signals via Gsα, resulting in the activation of the adenylyl cyclase/protein kinase A pathway [6, 7]. However, depending on its cellular localization and context, CRHR1 can activate multiple G proteins, which can trigger alternative second messengers [8]. For example, the coupling of CRHR1 to Gsα can activate the extracellular signal-regulated kinases 1 and 2 (ERK1/2) signaling pathway, which can also be activated by Gqα [9–11]. Furthermore, the coupling of CRHR1 to Gsα can result in intracellular calcium mobilization via the activation of AC, which activates the ɛ isoform of phospholipase C (PLCɛ) [12]. Up to now CRHR1 interactions with G proteins, arrestins, and G protein-coupled receptor kinases have extensively been studied [13, 14], whereas its interactions with other accessory or GPCR-interacting proteins, which would provide further specificity to CRHR1 signaling or determine the activation of particular downstream pathways, are largely unknown.

Membrane-associated guanylate kinases (MAGUKs) are synaptic scaffold proteins that are important in the assembly of receptors and intracellular signaling proteins [15]. MAGUKs comprise PSD95/discs large/zona occludens 1 (PDZ) domains and enzymatically inactive guanylate kinase (GuK)-like domains, and they commonly contain SRC homology 3 (SH3) domains. The PSD95 and MAGI subfamilies are MAGUKs that represent crucial components of the excitatory post-synaptic density [16], but they are also located at non-synaptic sites, e.g. SAP102 and PSD95 are associated with extrasynaptic NMDA receptors [17]. MAGUKs can bind to many surface receptors via their PDZ domains, which directly interact with the receptors’ C-terminal PDZ binding motif [15].

In this study, we identified and characterized new interacting partners of CRHR1 and confirmed previously reported ones, belonging to the MAGUK family. We were able to demonstrate the co-expression of CRHR1 and interacting partners in neurons of the murine brain. CRHR1 was present throughout the neuronal plasma membrane, including the excitatory post synapse, where it co-localized with MAGUKs. We functionally proved that the amino acid sequence S^412^-T^413^-A^414^-V^415^ at the C-terminus of CRHR1 represents a valid class I PDZ binding motif and specified the PDZ domains of the interacting partners that mediate the interaction with this C-terminal receptor motif. Moreover, the interactions were responsible for the clustering of CRHR1 with individual MAGUKs in HEK293 cells.

Altogether, our study identified new and confirmed previously reported interacting partners of CRHR1, shedding light on CRHR1 signaling pathways. This is important for the design of new CRHR1-based pharmacological approaches for interferences with stress-related disorders.

## Materials and Methods

### Plasmid constructs

The myc-GFP-CRHR1 and PSD95-flag expression plasmids were constructed as follows: CRHR1 and PSD95 cDNAs were amplified by PCR from a murine brain cDNA library and cloned into pcDNA3 (Invitrogen). For myc-GFP-CRHR1, the signal sequence of larval cuticle protein 3 (LCP3; GenBank accession number NM_057273), followed by a c-myc tag (provided by Rolf Sprengel (MPI for Medical Research, Heidelberg, Germany) was inserted in frame at the N-terminus of EGFP [18]. For myc-GFP-CRHR1-STAVA, appropriate oligonucleotides were used to design a linker, which was inserted between the *Bsp*EI and *Xho*I sites of the wild-type (WT) CRHR1 construct to add the additional alanine at the C-terminus. Furthermore, flag-CRHR1 was kindly provided by Felix Hausch (MPI of Psychiatry, Munich, Germany) and comprised the hemagglutinin signal sequence followed by a flag-tag and the human CRHR1 cDNA (NM_004382) cloned into pcDNA3. flag-CRHR1-STAVA was then cloned using site-directed mutagenesis (QuikChange Lightning, Agilent Technologies) based on the flag-CRHR1 construct. HA-CRHR1 was generated using appropriate oligonucleotides to replace the flag tag of flag-CRHR1 with a HA tag. myc-CRHR1 mutants with the C-terminal amino acid sequences STAA, SAAV, ATAV and STAVA were designed using appropriate primers for PCR amplification. myc-GFP-CRHR1 mutants with the C-terminal amino acid sequences SEAV and ETAV were cloned using site-directed mutagenesis. Murine PSD95 was tagged with a C-terminal flag epitope. PSD95 PDZ1-flag, PSD95 PDZ2-3-flag, PSD95 PDZ3-flag, and PSD95 ΔPDZ1-3-flag were cloned by using corresponding primers for PCR amplification using PSD95 WT as a template. PSD95 PDZ1-3 comprises amino acids (aa) 40–498 of WT PSD95. PSD93-GFP (rat) in pcDNA3 was kindly provided by Damian Refojo (MPI of Psychiatry, Munich, Germany). HA-SAP97 (rat) and the mutants HA-SAP97 PDZ1-3, HA-SAP97 PDZ1-2, and HA-SAP97 PDZ1 were provided by Daniela Gardiol (Instituto de Biologia Molecular y Celular de Rosario, Rosario, Argentina) [19]. Murine flag-SAP102 was kindly provided by Robert Zalm (VU University Amsterdam, The Netherlands) [20], and the mutant flag-SAP102 PDZ3 (aa 394–849) was generated by excision of PDZ1-2 with *Eco*RI and *Bbv*CI and subsequent religation using an appropriate linker. Syntenin-1-myc was kindly provided by Pascale Zimmermann (University of Leuven and Flanders Interuniversity Institute for Biotechnology, Leuven, Belgium). myc-MAGI2 (rat) and the mutants myc-MAGI2 WW + PDZ1, myc-MAGI2 PDZ2-5, and myc-MAGI2 PDZ0 + GuK were kindly provided by Nils Brose (MPI for Experimental Medicine, Göttingen, Germany) [21].

### Yeast-two-hybrid screen

The yeast two-hybrid screen was performed using the matchmaker system according to the manufacturer’s recommendations (Clontech). In brief, a cDNA fragment encoding the C-terminus of murine and human CRHR1 (aa 368–415; NM_007762) was inserted into pGBKT7 (Clontech) at *Eco*RI/*Bam*HI sites (pGBKT7-RC) and transformed into the yeast strain AH109. The resulting strain was mated with yeast strain Y187, which was transformed with a mouse cDNA brain library that was fused to the Gal4 activation domain in pGADT7 (Clontech). Mating proceeded for 24 h, and the mated yeast was plated on quadruple dropout medium SD/-Ade/-His/-Leu/-Trp plus X-β-Gal 25 mg/l to select colonies containing both plasmids and identify positive interaction partners. During incubation at 30°C for 7 days, the mating was checked for the appearance of new colonies every day. The cDNA of yeast colonies was isolated and identified by sequencing. The full-lengths DNA was then used for further experiments, except stated otherwise.

### Automated yeast-two-hybrid screen

The second Y2H screen was performed using an automated interaction mating procedure [22]. The pBTM-D9 plasmid encoding a C-terminal fragment of CRHR1 (for the production of a LexA domain-containing bait protein) was transformed into the *L40ccua* MATa yeast strain. For interaction screening, yeast clones expressing non-self-activating bait proteins were pooled and mated against an arrayed library of MATα yeast clones, expressing 16,888 human prey proteins with a Gal4 activation domain using pipetting and spotting robots. The automated Y2H screens were repeated 4-times to obtain a high coverage of protein-protein interactions [22]. Positive clones with activated *HIS3* and *URA3* reporter genes were identified through spotting of yeast strains onto selective plates. Prey proteins that interact with CRHR1 were finally identified by a second automated interaction-mating step, allowing the deconvolution of initially pooled bait proteins. The second interaction mating step was repeated five times to increase the coverage of protein interaction data. The cDNA of yeast colonies was isolated and identified by sequencing.

### Animals

Mice were housed under standard laboratory conditions (22 ± 1°C, 55% ± 5% humidity) with food and water ad libitum. Animal experiments were conducted in accordance with the Guide for the Care and Use of Laboratory Animals of the Government of Upper Bavaria (Germany) and approved by the Animal Care and Use Committee of the Max Planck Institute of Psychiatry (Munich, Germany). For in situ hybridization (ISH), brains were dissected from 2–3-month-old male mice sacrificed by an overdose of isoflurane. For single ISH, brains of WT mice were used. For double ISH, brains of CRHR1-GFP reporter mice [23] were used. For adeno-associated virus (AAV)-mediated expression of CRHR1-WT or CRHR1-STAVA, primary neurons from heterozygous Nex-Cre mice [24] were prepared.

### Single in situ hybridization

Single in situ hybridization (ISH) was conducted as previously described [5]. The following riboprobes were used: CRHR1, nucleotides 1728–2428 of GenBank accession no. NM_007762; PSD95 nucleotides 572–1302 of NM_007864; PSD93, nucleotides 243–754 of NM_011807; SAP97, nucleotides 27–1124 NM_001252436; SAP102, nucleotides 3883–4788 of NM_016747; and MAGI2, nucleotides 5336–5958 of NM_001170746. For comparison a false color display was applied on the basis of the autoradiography images.

### Double in situ hybridization

To detect co-localization at the single-cell level, double ISH was performed as previously described [5]. The riboprobes of ISH and additionally for GFP nucleotides 1757–2388 of JX679622 were used. Hybridization was performed overnight with a riboprobe concentration of 8.5 × 10^6^ cpm μl^-1^.

### Quantitative real time PCR

To detect mRNA levels, quantitative real time PCR was conducted as previously described [25]. The following primers were used: CRHR1: fwd. 5′-GGG-CCA-TTG-GGA-AAC-TTT-A-3′, rev. 5′-ATC-AGC-AGG-ACC-AGG-ATC-A-3′, NM_007762; CRH: fwd. 5′-GA-GGC-ATC-CTG-AGA-GAA-GTC-C-3′, rev. 5′-TGT-TAG-GGG-CGC-TCT-CTT-C-3′, NM_205769; CRHR2: fwd. 5′-TGT-GGA-CAC-TTT-TGG-AGC-AG-3′, rev. 5′-TGC-AGT-AGG-TGT-AGG-GAC-CTG-3′, NM_009953; MAGI2: fwd. 5′-CAC-GTC-CCG-GAG-TAT-GGA-3′, rev. 5′-TGC-TTG-TCA-CTT-TTC-ATG-CAC-3′, NM_001170746; PSD93: fwd. 5′-CAA-TCA-GAA-ACG-CTC-CCT-GT-3′, rev. 5′-CCC-ACT-GTC-CTT-GCT-CTT-GT-3′, NM_011807; PSD95: fwd. 5′-TCT-GTG-CGA-GAG-GTA-GCA-GA-3′, rev. 5′-CGG-ATG-AAG-ATG-GCG-ATA-G-3′, NM_007864; SAP97: fwd. 5′-TTT-CCC-GAA-AAT-TTC-CCT-TC-3′, rev. 5′-TGG-CAT-TAG-AAG-TTA-CGT-GCT-G-3′, NM_007862; SAP102: fwd. 5′-GGG-CCA-GTT-CAA-TGA-TAA-TCT-C-3′, rev. 5′-CGT-TGC-CGG-AGA-CAT-CTA-AG-3′, NM_001177779.

### Fluorescence polarization assay

The fluorescence polarization assay was performed as recently described [26]. In brief, a competition setup was used in which a constant concentration of isolated PSD95 PDZ domains and labeled reference ligands were titrated with the C-terminal 10 amino acids of CRHR1. Three Cy5-labeled peptides with well-documented interactions with PSD95 were used as reference ligands for the competition assay: Cy5-KIF1Bα for PDZ1, Cy5-GluN2B for PDZ2 and PDZ1-2, and Cy5-CRIPT for PDZ3.

### Cell culture and transfection

HEK293 cells were maintained in DMEM (Invitrogen) supplemented with 10% FCS and 1% penicillin/streptomycin (growth medium). For transfection, HEK293 cells were plated in antibiotic-free DMEM and transfected with Lipofectamine 2000 (Invitrogen) according to the manufacturer´s protocol. After 5–6 h, the medium was changed to normal growth medium. Primary hippocampal cultures were prepared from embryonic day 17–18 mouse brains and grown in Neurobasal A medium supplemented with B27 (Invitrogen) and GlutaMAXI (Invitrogen) as recently described [26]. Neurons were plated on coverslips (Menzel) coated with 50 μg/ml poly- d-lysin (Sigma) and 5 μg/ml laminin (Invitrogen) at a density of 65,000 cells per coverslip.

### Production of rAAV

To produce AAV vectors expressing myc-GFP-CRHR1 and myc-GFP-CRHR1-STAVA the respective fragment was cloned with *Nhe*I and *Asc*I into pAAV-EF1a-DIO-EYFP-WPRE provided by Karl Deisseroth and Charu Ramakrishnan (Stanford University). This approach allows Cre recombinase-mediated induction of CRHR1 expression. AAV8Y733F-pseudotyped single-strand AAV2 vectors were produced as described recently [27]. In brief, 293T cells were transfected with the trans plasmids pAdDeltaF6, pAAV2/8Y733F, and a cis plasmid (pAAV-myc-GFP-CRHR1WT or STAVA) using the calcium phosphate method. rAAV2/8Y7333F particles were harvested after 48 h followed by iodixanol-gradient purification. The 40%–60% iodixanol interface was further purified and concentrated by ion exchange chromatography on a 5 ml HiTrap Q Sepharose column using an ÄKTA Basic FPLC system (GE Healthcare, Munich, Germany) according to previously described procedures, followed by further concentration using Amicon Ultra-4 Centrifugal Filter Units (Millipore, Schwalbach, Germany). Physical titers [in genome copies (gc)/ ml] were determined by quantitative PCR on a LightCycler 480 (Roche Applied Science, Mannheim, Germany) using KAPPA SYBR FAST kit (Peqlab, Erlangen, Germany) and the following primer set: WPREF: 5′-AGT-TGT-GGC-CCG-TTG-TCA-GG-3′ and WPRER: 5′-AGT-TCC-GCC-GTG-GCA-ATA-GG-3′. For myc-GFP-CRHR1 and myc-GFP-CRHR1-STAVA, 1.2 × 10^12^ and 3 × 10^12^ gc/ ml, respectively, were achieved.

### Immunocytochemistry

To detect a protein of interest, immunocytochemistry was conducted as previously described [5]. In brief, neurons after 20 days in culture or HEK293 cells 24 h after transfection were fixed with 4% PFA containing 4% sucrose for 30 min, washed three times with PBS and permeabilized with 0.1% Triton X-100 in PBS three times for 5 min each. After the cells were washed with PBS for 5 min, they were blocked with 0.1% Triton X-100 in PBS containing 5% BSA for 1 h and additionally washed two times. Cells were incubated overnight at 4°C with the respective antibody: anti-GFP (ab13970, Abcam), anti-flag (F3165, Sigma–Aldrich), anti-HA (C29F4, Cell Signaling), anti-myc (sc798B, Santa Cruz), anti-CRHR1 (sc1757, Santa Cruz), anti-PSD95 (75–028, UCDavis/ NIH NeuroMab Facility), anti-gephyrin (147111, Synaptic Systems), anti-synapsin (106001, Synaptic Systems), anti-MAP2 (ab5622, Abcam), anti-SAP97 (PA1-741, Thermo Fisher Scientific), and anti-ankyrin G (75–147, UCDavis/ NIH NeuroMab Facility). Subsequently, cells were washed three times with PBS and afterwards incubated with a secondary antibody conjugated with Alexa Fluor 594 (anti-rabbit, A11037, Invitrogen), Alexa Fluor 488 (anti-chicken, A11039, Invitrogen), Alexa Fluor 594 (anti-goat, A11058, Invitrogen), Alexa Fluor 594 (anti-mouse, A11032, Invitrogen), Alexa Fluor 488 (anti-rabbit, A11034, Invitrogen) or Alexa Fluor 488 (anti-mouse, A11029, Invitrogen). After secondary antibody treatment, cells were washed for 5 min three times, stained with DAPI for 5 min, and mounted using VectaShield medium (Vector). Labeled cells were imaged by laser-scanning confocal microscopy using a 40× and 60× objective with 4 times zoom. Images were then analyzed using FLUOVIEW (FV10-ASW, Version 2.0a), Image J, and Adobe Photoshop CS2.

### Co-immunoprecipitation

At 24–48 h post transfection, cells seeded onto 6-well plates or 10 cm dishes were lysed in lysis buffer (50 mM Tris HCl pH 8, 150 mM NaCl, 0.1% SDS, 1% Triton X-100, 1 mM EDTA pH 8, protease inhibitors) for 1 h on a rocking platform at 4°C. Lysates were scrape-collected and centrifuged for 20 min at 40,000 ×g. Clarified lysates were pre-cleared by incubation with Dynabeads Protein G (#100-04D, Life Technologies) for 30 min. Similar protein amounts assessed in a test Western blot were used for overnight incubation at 4°C with the appropriate antibody, i.e. anti-GFP (ab6556, Abcam), anti-myc (ab9106, Abcam), anti-HA (C29F4, Cell Signaling), or anti-flag (F3165, Sigma–Aldrich). Lysates with antibody were incubated with pre-equilibrated Protein G Dynabeads for 4 h at 4°C rotating. The beads were washed 3 × 10 min with washing buffer I (50 mM Tris HCl pH 8, 500 mM NaCl, 0.1% SDS, 0.5% Triton X-100, 1 mM EDTA pH 8) and 1 × 10 min with washing buffer II (50 mM Tris HCl pH 7.4, 0.1% SDS, 0.5% Triton X-100, 1 mM EDTA pH 8). For elution beads were incubated with 1× Roti-Load (Roth) at 37°C for 30 min or at 95°C for 10 min. Samples were separated by 10% SDS-PAGE gels and transferred to Immobilon-P PVDF membranes (Millipore) and immunoblotted as indicated in the figures. The first antibody was incubated overnight and the second antibody anti-rabbit (#7074, Cell Signaling) or anti-mouse (#7076, Cell Signaling) for 2 h.

## Results

### Identification of MAGUKs as candidate interaction partners of CRHR1

To identify interaction partners of CRHR1, we performed a yeast two-hybrid (Y2H) screen using the C-terminal cytoplasmic tail (aa 368–415) of murine/rat CRHR1 (data not shown). As the most abundant candidate interaction partners, we identified members of the MAGUK family: PSD95, SAP97, SAP102, and MAGI2. We included PSD93 in our further studies because of its high homology to the other members of the PSD95 subfamily of MAGUKs identified in our Y2H screen.

### Co-expression of CRHR1 and candidate interaction partners

**Fig 1:**
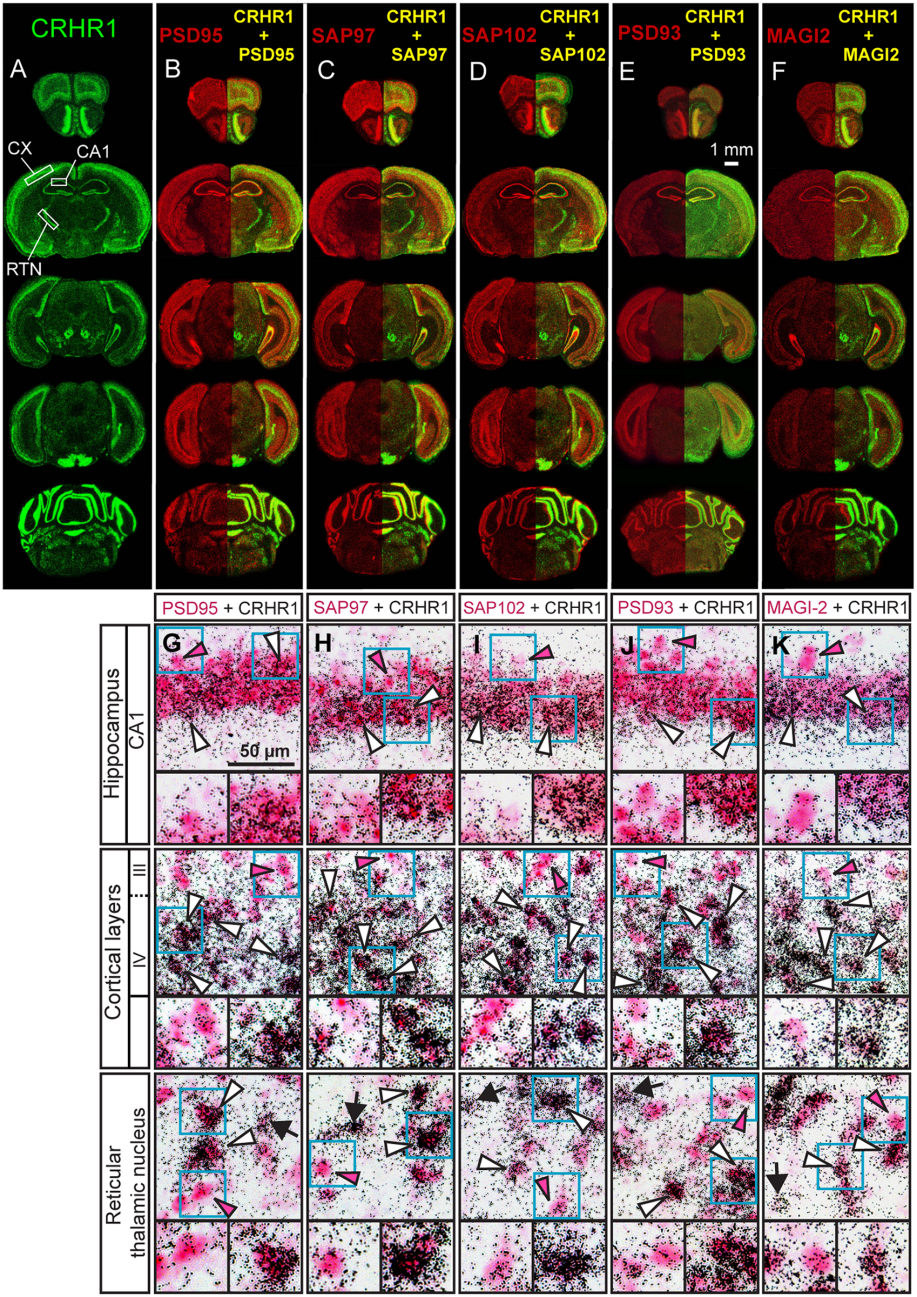
CRHR1 and candidate MAGUKs are co-expressed in the adult mouse brain. (A–F) Expression of mRNA of CRHR1, PSD95, SAP97, SAP102, PSD93, and MAGI2 was assessed by single *in situ* hybridization on consecutive coronal brain sections of wild-type mice. For comparison, representative photomicrographs of autoradiographs are shown in a false color display. CRHR1 expression is displayed in green, and PSD95, SAP97, SAP102, PSD93, and MAGI2 expression are displayed in red. The yellow signal reveals brain structures of overlapping expression of CRHR1 with respective MAGUKs. (G–K) Double *in situ* hybridization revealed co-expression of CRHR1 and interacting partners at the cellular level. Depicted are representative photomicrographs of coronal brain sections of CRHR1-GFP reporter mice. Blue boxes depict individual neurons shown below as magnifications (left: single MAGUK positive neuron, right: MAGUK and CRHR1 double positive neuron). Arrows indicate single positive cells stained by silver grains for CRHR1, and red arrowheads indicate cells expressing the candidate MAGUK only. White arrowheads indicate double-positive cells co-expressing CRHR1 and its respective interaction partners. CA: cornu ammonis, RTN: reticular thalamic nucleus.

As a first step toward the validation of the identified interactions we assessed whether CRHR1 and the potential interaction partners are co-expressed in the mouse brain which would be a prerequisite for a direct physical interaction. Expression was analyzed via single and double *in situ* hybridization (ISH) in the adult mouse brain (Fig 1). Single ISH with specific riboprobes for CRHR1 and respective MAGUKs revealed their spatial distribution in the mouse brain. CRHR1 exhibited strong co-localization with interacting partners in brain regions such as the olfactory bulb, cortex, hippocampus and cerebellum (Fig 1A–1F). We next assessed co-expression on the cellular level using double ISH (DISH). To enhance the endogenous CRHR1 signal and facilitate co-localization, we used CRHR1-GFP reporter mice, which overexpress GFP specifically in CRHR1-positive neurons [23]. DISH revealed that the mRNA of CRHR1 and candidate interaction partners clearly co-localized in pyramidal neurons of the hippocampal CA1 region and the cortex as well as in inhibitory neurons of the reticular thalamic nucleus (Fig 1G–1K).

**Fig 2:**
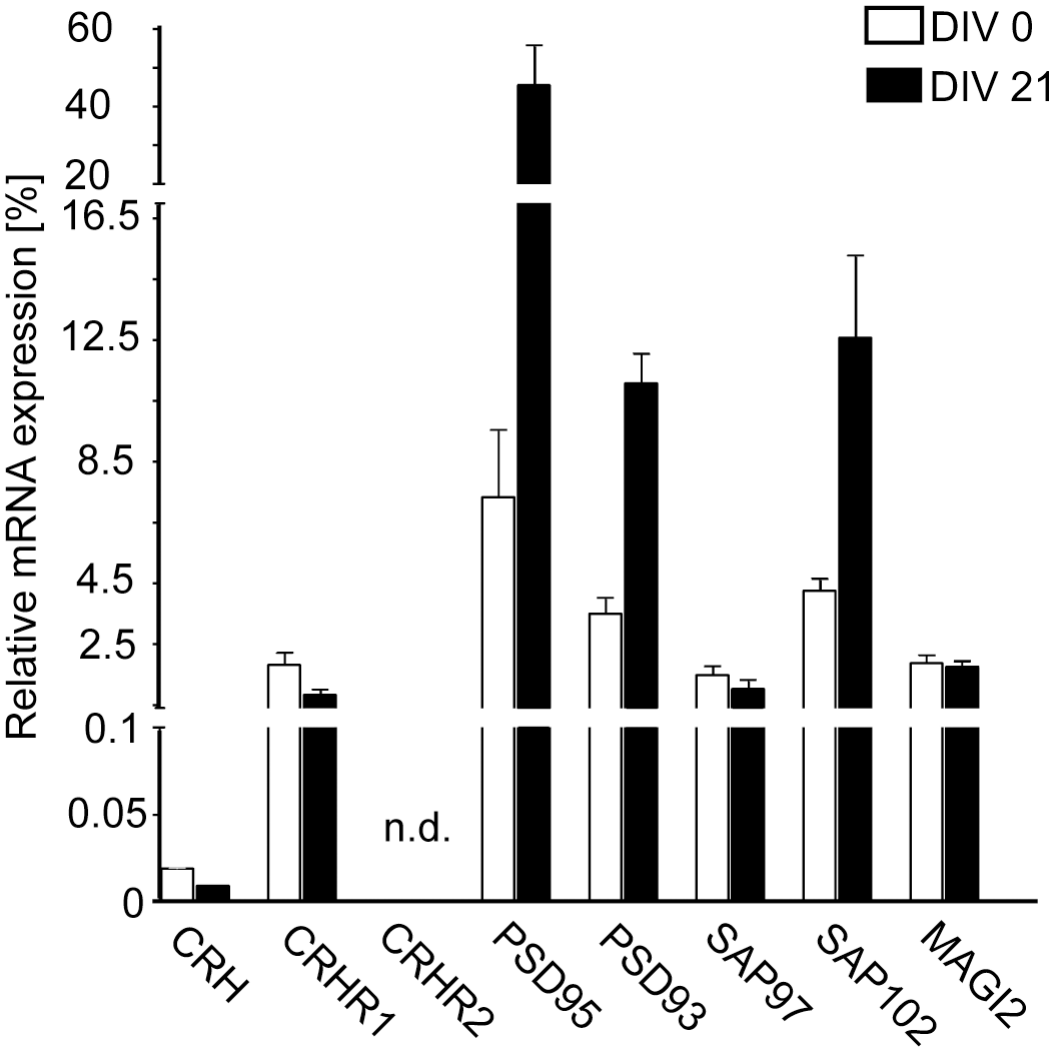
Expression of CRHR1 and its interacting MAGUKs in primary hippocampal neurons. The mRNA levels of CRHR1, its ligand CRH, the related receptor CRHR2 and the candidate partners PSD95, SAP97, SAP102, PSD93, and MAGI2 were determined by quantitative real time PCR and normalized to ribosomal protein L19 (RPL-19) mRNA expression. Expression levels were analyzed at DIV 0 (days in vitro) and DIV 21. n.d.: not detected.

In addition, we prepared primary hippocampal neurons and determined the expression of CRHR1 and MAGUKs by quantitative real time PCR. We were able to detect the mRNA expression of CRHR1 and its ligand CRH, but not CRHR2, in primary neurons (Fig 2). Candidate interaction partners PSD95, SAP97, SAP102, PSD93, and MAGI2 were also detected in cultured neurons. PSD95, SAP102, and PSD93 were significantly up-regulated from DIV 0 (days in vitro) to DIV 21.

Altogether, the clear demonstration of co-expression with CRHR1 supports the possibility of interactions with the identified MAGUKs.

### Functional assessment of the C-terminal CRHR1 PDZ-binding motif

**Fig 3:**
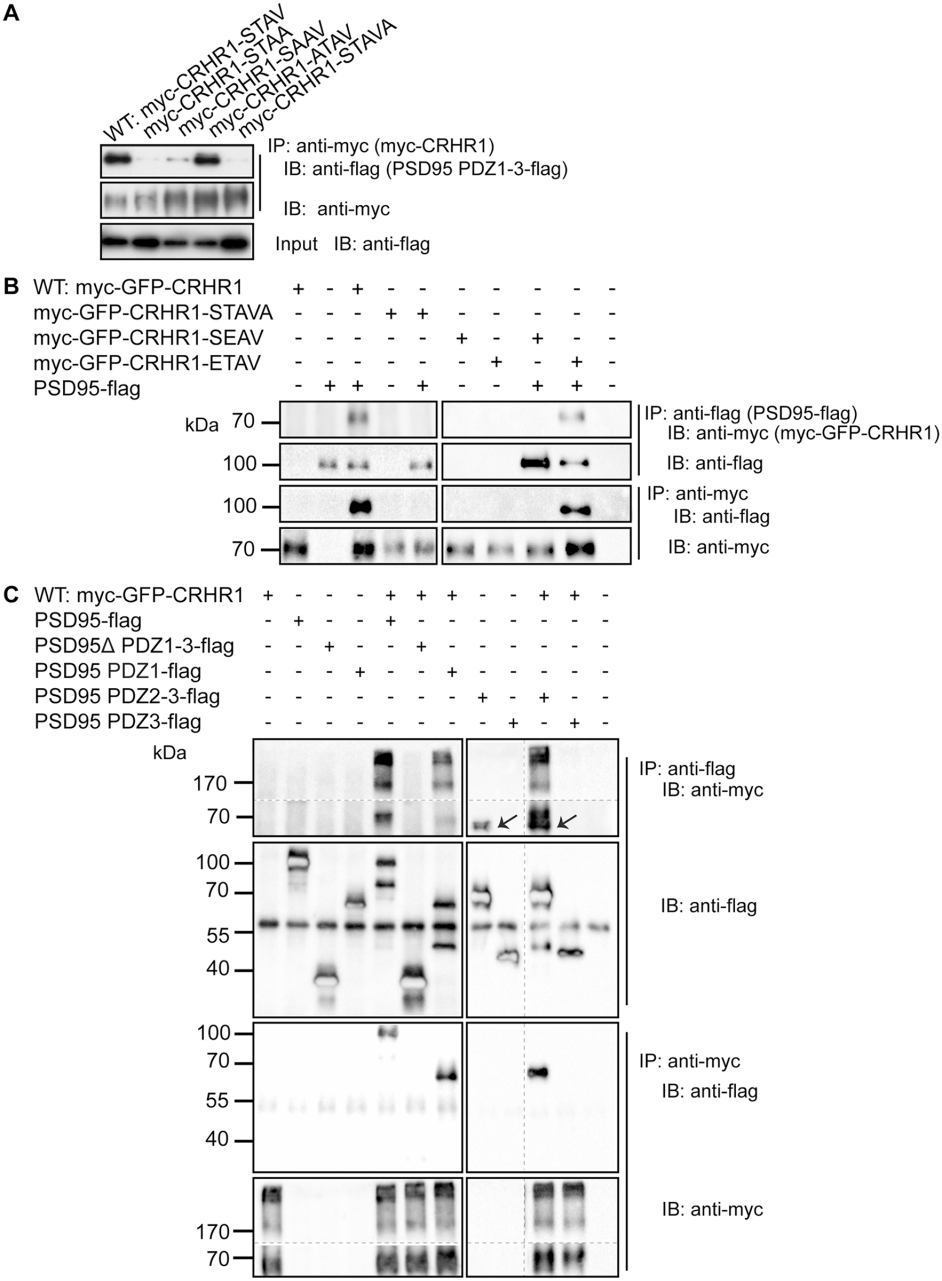
PDZ binding motif-mediated interaction of CRHR1 with PSD95 depends on PDZ1 and PDZ2. Co-immunoprecipitations (Co-IPs) were performed using lysates of HEK293 cells transiently transfected as indicated. (A) PSD95 PDZ1-3-flag was co-immunoprecipitated with wild-type myc-CRHR1-STAV and to variable degrees with myc-CRHR1-STAA, myc-CRHR1-SAAV, and myc-CRHR1-ATAV but not with the myc-CRHR1-STAVA mutant. (B) PSD95-flag was co-immunoprecipitated with myc-GFP-CRHR1 but not with myc-GFP-CRHR1-STAVA. Accordingly, myc-GFP-CRHR1, but not myc-GFP-CRHR1-STAVA, was co-immunoprecipitated with PSD95. PSD95-flag was not co-immunoprecipitated with the phospho-mimicking variant myc-GFP-CRHR1-SEAV, but with the phospho-mimicking variant myc-GFP-CRHR1-ETAV. Accordingly, myc-GFP-CRHR1-SEAV was not co-immunoprecipitated with PSD95-flag, but myc-GFP-CRHR1-ETAV was co-immunoprecipitated with PSD95-flag. (C) myc-GFP-CRHR1 was co-immunoprecipitated with the PSD95 mutants PSD95 PDZ1-flag and PSD95 PDZ2-3-flag, but not with PSD95Δ PDZ1-3-flag or PSD95 PDZ3-flag. Complementary results were obtained when the Co-IP was performed against myc-GFP-CRHR1. In lanes 8 and 10 a slight cross-reactivity with PSD95 PDZ2-3-flag (→)—appearing as a band below myc-GFP-CRHR1—was observed. Dashed lines indicate that the samples were run on the same immunoblot (IB), however, not in adjacent lanes. Continuous lines separate different IBs from the same experiment. The ~55 kDa band in the anti-flag IB represents the heavy chain of the primary antibody. IP: immunoprecipitation.

As recently proposed [28], the amino acid sequence S^412^-T^413^-A^414^-V^415^ at the C-terminus of murine and human CRHR1 resembles a C-terminal class I PDZ-binding motif that in general has the consensus sequence S/T-X-Φ, where Φ represents a bulky hydrophobic residue. For the validation and detailed characterization of the interplay with potential interactors, we generated C-terminal CRHR1 mutants with a functionally disrupted PDZ binding motif. Interactions were probed by co-immunoprecipitation (Co-IP) using lysates of HEK293 cells transiently co-transfected with CRHR1 and MAGUK variants. First, we tested the capacity of CRHR1 mutants to co-immunoprecipitate PSD95 PDZ1-3 (Fig 3A, lanes 2–5), which was originally identified in the Y2H screen and comprises the three PDZ domains and the SH3 domain of PSD95. PSD95 PDZ1-3 was readily co-immunoprecipitated with WT CRHR1 (Fig 3A, lane 1). Moreover, we identified that the CRHR1-STAVA mutant, which contains an additional alanine at the C-terminus, most efficiently disrupted the interaction of CRHR1 with PSD95 PDZ1-3 (Fig 3A, lane 5). Subsequently we demonstrated that also the full-length PSD95 interacted with CRHR1, as indicated by the successful Co-IPs in both directions (Fig 3B, lane 3); i.e., PSD95 was detected in the Western blot (WB) following immunoprecipitation (IP) against CRHR1, and CRHR1 was detected in the WB following an IP against PSD95. However, the CRHR1-STAVA mutant carrying a functionally impaired PDZ binding motif did not interact with PSD95 (Fig 3B, lane 5). A major advantage of this particular mutant compared, for example, to the deletion of the entire PDZ binding motif is the fact that it does not interfere with S^412^ and T^413^ which are potential GRK or PKC phosphorylation sites [7]. Therefore, we used the CRHR1-STAVA mutant in subsequent experiments in addition to the wild-type receptor (CRHR1-WT) to further characterize the interaction of CRHR1 with MAGUKs.

To investigate the effect of phosphorylation on the binding capacity of the PDZ binding motif, we substituted potential phosphorylation sites upstream (Ser412) or within (Thr413) the PDZ binding motif by phospho-mimicking glutamic acid (E). We observed that the CRHR1-SEAV mutant, which contains at position 413 within the PDZ binding motif a glutamic acid instead of threonine, efficiently disrupted the interaction of CRHR1 with PSD95 as demonstrated via Co-IPs against CRHR1 and PSD95, respectively (Fig 3B, lane 8). Moreover, the CRHR1-ETAV mutant containing a phospho-mimicking glutamic acid adjacent to the PDZ binding motif did not alter the interaction capability of CRHR1 with PSD95 (Fig 3B, lane 9).

### Characterization of the interaction of CRHR1 with PSD95

We then investigated PSD95 mutants to further specify the PDZ domains relevant for the interaction with the CRHR1 PDZ binding motif. Co-IPs against CRHR1 and against PSD95 mutants revealed interactions of CRHR1 with PSD95 PDZ1-flag (Fig 3C, lane 7) and PSD95 PDZ2-3-flag (Fig 3C, lane 10). By contrast, no interaction of CRHR1 was detected with PSD95Δ PDZ1-3-flag (Fig 3C, lane 6) or PSD95 PDZ3-flag (Fig 3C, lane 11).

To further determine the affinity of the CRHR1 PDZ binding motif to individual PDZ domains of PSD95 by different means, we performed a fluorescence polarization assay [26]. We used the last 10 amino acids of CRHR1 and the three PDZ domains of PSD95 in a competition set-up in which a constant concentration of respective PDZ domain and a labeled reference ligand were titrated against the unlabeled CRHR1 peptide. We identified the following order of binding affinities in the FP assay: PDZ2 (K_i_ 19 ± 0.4 μM) > PDZ1-2 (K_i_ 24 ± 0.4 μM) > PDZ3 (K_i_ 48 ± 6 μM) > PDZ1 (K_i_ 77 ± 8 μM). These results underscore the particular importance of the PDZ2 domain for the interaction between PSD95 and CRHR1.

### Characterization of the interaction of CRHR1 with SAP97

**Fig 4:**
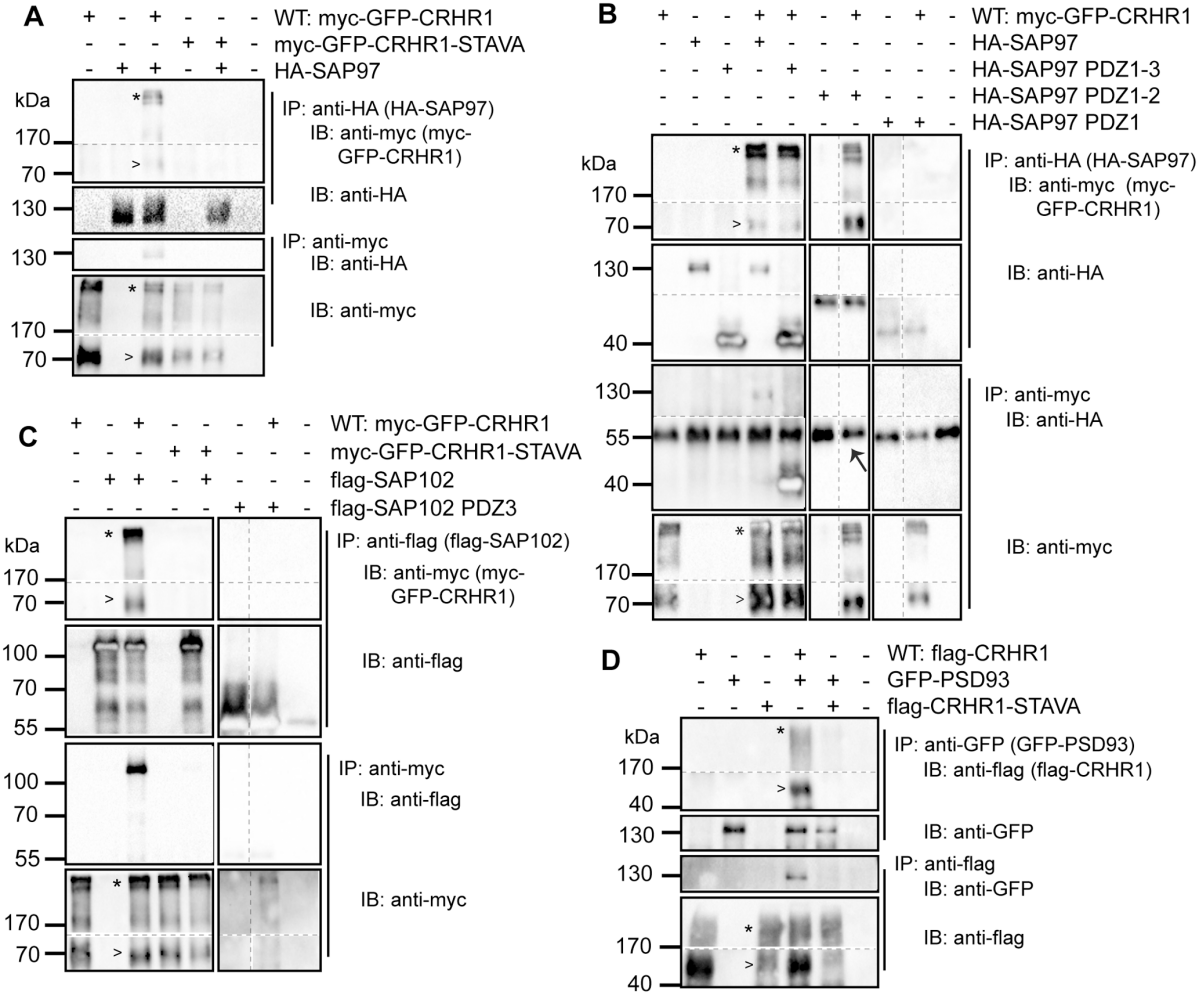
CRHR1 interaction with SAP97, SAP102 and PSD93 depends on the PDZ binding motif. Co-IPs were performed using lysates of HEK293 cells transiently transfected as indicated. (A) myc-GFP-CRHR1 but not myc-GFP-CRHR1STAVA was co-immunoprecipitated with HA-SAP97, and similarly, HA-SAP97 was co-immunoprecipitated with myc-GFP-CRHR1 but not with myc-GFP-CRHR1-STAVA. (B) HA-SAP97 PDZ1-3 and HA-SAP97 PDZ1-2 were co-immunoprecipitated with myc-GFP-CRHR1 and accordingly, these SAP97 variants were co-immunoprecipitated with myc-GFP-CRHR1 using an anti-myc antibody in the Co-IP. No interaction was observed for HA-SAP97 PDZ1. HA-SAP97 PDZ1-2 detected by the anti-HA antibody following the anti-myc Co-IP has the same molecular weight and thus is indistinguishable from the heavy chain of the anti-myc antibody (→). (C) myc-GFP-CRHR1 but not myc-GFP-CRHR1-STAVA was co-immunoprecipitated with flag-SAP102. Similarly, flag-SAP102 was co-immunoprecipitated with myc-GFP-CRHR1 but not with myc-GFP-CRHR1-STAVA. Moreover, flag-SAP102 PDZ3 was not detected following an immunoprecipitation (IP) of myc-GFP-CRHR1. (D) flag-CRHR1 but not flag-CRHR1-STAVA was co-immunoprecipitated with GFP-PSD93 and accordingly, GFP-PSD93 was co-immunoprecipitated with flag-CRHR1 but not with flag-CRHR1-STAVA. CRHR1 always showed high molecular weight complexes (*) together with the monomeric form (>). Therefore, high molecular weight complexes are also shown when necessary. Dashed lines indicate that the samples were run on the same immunoblot (IB), however, not in adjacent lanes. Continuous lines separate different IBs from the same experiment. (B) The ~55 kDa band represents the heavy chain of the primary antibody.

Co-IPs against CRHR1 and SAP97, respectively, disclosed that the full-length HA-SAP97 directly interacted with CRHR1 (Fig 4A, lane 3) but not with myc-GFP-CRHR1-STAVA (Fig 4A, lane 5). Co-IPs further revealed that the mutants HA-SAP97 PDZ1-3 (Fig 4B, lane 5) and HA-SAP97 PDZ1-2 (Fig 4B, lane 7) interacted with CRHR1. However, no interaction was detected with HA-SAP97 PDZ1 (Fig 4B, lane 9). This result indicates that CRHR1 interacts via its PDZ binding motif with PDZ2 but we cannot exclude that it may also interact with PDZ3 of SAP97.

### Characterization of the interaction of CRHR1 with SAP102

Co-IPs against CRHR1 and SAP102 revealed an interaction in both directions (Fig 4C, lane 3) of SAP102 with CRHR1 via the PDZ binding motif (Fig 4C, lane 5). In contrast, the mutant SAP102 PDZ3-flag did not interact with CRHR1 (Fig 4C, lane 7) confirming that PDZ1 and PDZ2 domains of SAP102 are essential for the interaction.

### CRHR1 interacts with PSD93 via the PDZ binding motif

We further tested PSD93, which was also co-immunoprecipitated with CRHR1. Similarly, CRHR1 was co-immunoprecipitated with PSD93 (Fig 4D, lane 4). Furthermore, the PDZ binding motif inactive mutant CRHR1-STAVA clearly demonstrated that the interaction was conveyed by the PDZ binding motif (Fig 4D, lane 5).

### Characterization of the interaction of CRHR1 with MAGI2

**Fig 5:**
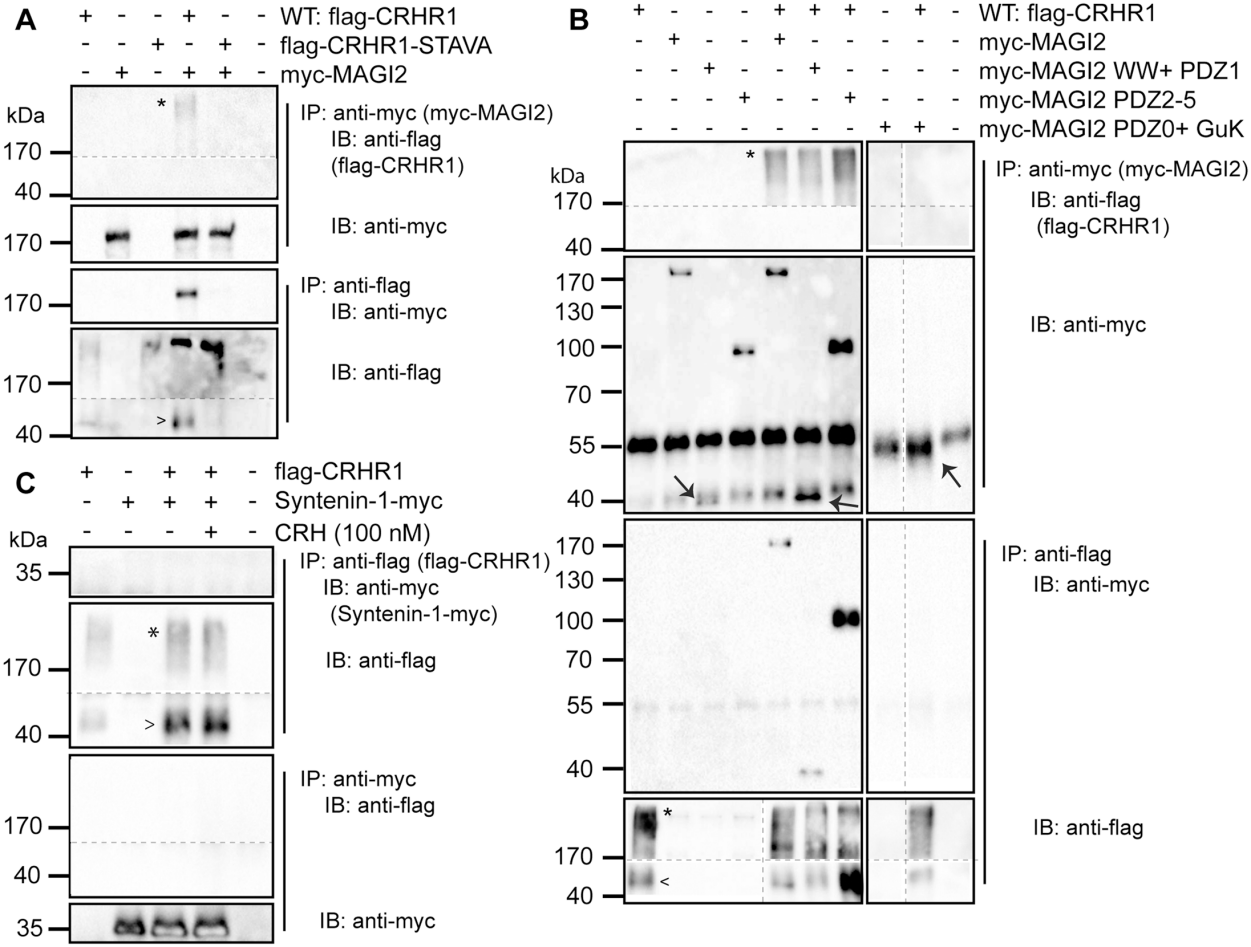
CRHR1 interaction with MAGI2 depends on the PDZ binding motif. Co-IPs were performed using lysates of HEK293 cells transiently transfected as indicated. (A) flag-CRHR1 but not flag-CRHR1-STAVA interacted with myc-MAGI2, and myc-MAGI2 was co-immunoprecipitated with flag-CRHR1 but not with flag-CRHR1-STAVA. (B) flag-CRHR1 was co-immunoprecipitated with myc-MAGI2, myc-MAGI2 WW + PDZ1, and myc-MAGI2 PDZ2-5 but not with myc-MAGI2 PDZ0 + GuK. Co-IPs were also successful in the other direction. The bands for the mutants of MAGI2 in the control blot are highlighted when necessary (→). (C) flag-CRHR1 neither interacted with syntenin-1-myc under basal conditions, nor after CRH (100 nM) treatment. Similarly syntenin-1-myc was not co-immunoprecipitated with flag-CRHR1. CRHR1 showed high molecular weight complexes (*) together with the momomeric form (>). Dashed lines indicate that the samples were run on the same immunoblot (IB), however, not in adjacent lanes. Continuous lines separate different IBs from the same experiment. The ~55 kDa band in the IB of anti-myc represents the heavy chain of the primary antibody. IP: immunoprecipitation.

MAGI2 was co-immunoprecipitated with CRHR1 (Fig 5A, lane 4) but not with CRHR1-STAVA (Fig 5A, lane 5). Accordingly, CRHR1-WT, but not CRHR1-STAVA, was co-immunoprecipitated with MAGI2. Co-IPs with different mutants of MAGI2 were conducted to determinate the relevant PDZ domains of MAGI2 interacting with the PDZ binding motif of CRHR1. Co-IPs against CRHR1 and MAGI2 mutants revealed an interaction of CRHR1 with myc-MAGI2 WW + PDZ1 and myc-MAGI2 PDZ2-5 (Fig 5B, lanes 6, 7). In contrast, CRHR1 did not interact with myc-MAGI2 PDZ0 + GuK (Fig 5B, lane 9). These results indicate that all or at least some of the PDZ1-5 domains of MAGI2 are responsible for the interaction with CRHR1.

**Fig 6:**
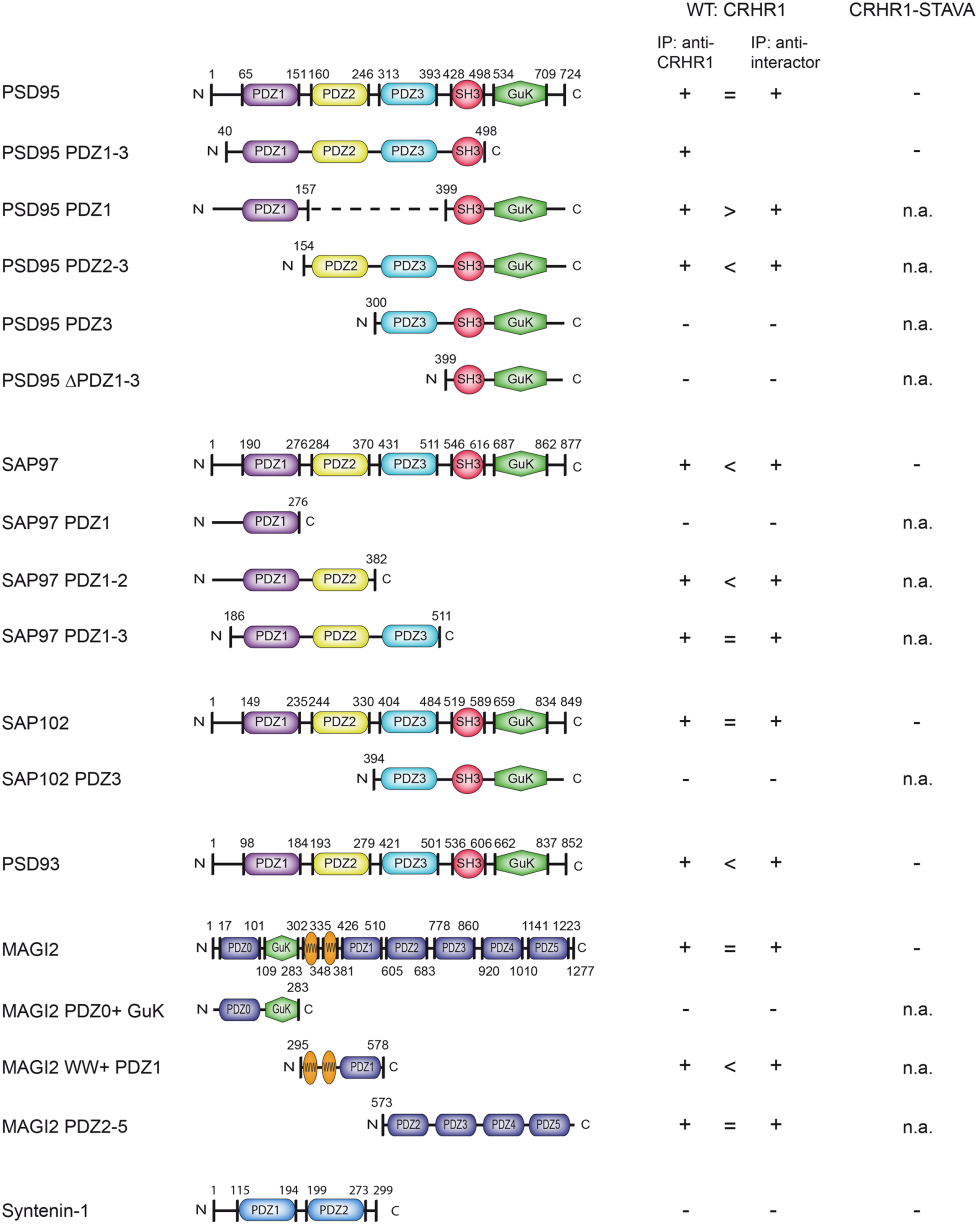
Summary of interactions of CRHR1 with MAGUKs, as revealed by co-immunoprecipitation (Co-IP). + interaction, = comparable Co-IP efficiency, > Co-IP more efficient when the immunoprecipitation (IP) was done against CRHR1, < Co-IP more efficient when the IP was done against interactor,—no interaction, n.a. not analyzed.

This PDZ-mediated interaction is highly specific as CRHR1 did not interact with the PDZ domain containing protein syntenin-1, which was also identified in an additional automated Y2H screen (Fig 5C, lane 3). In summary, the co-immunoprecipitation experiments revealed that the C-terminal CRHR1 PDZ binding motif interacts with different PDZ domains of MAGUKs (Fig 6).

### Co-localization of CRHR1-WT and CRHR1-STAVA with PSD95 and SAP97 in spines of primary hippocampal neurons

**Fig 7:**
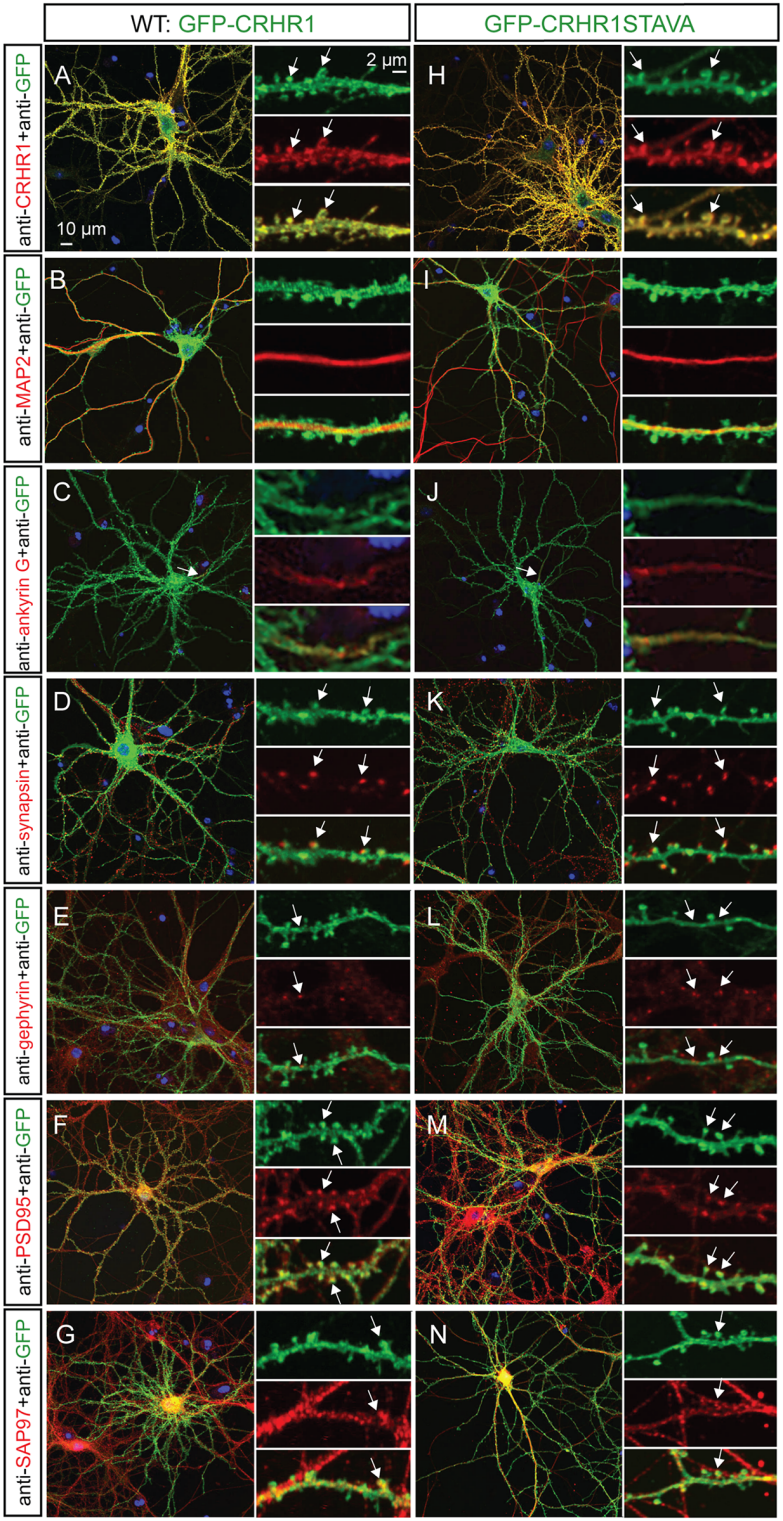
CRHR1-WT and CRHR1-STAVA localize throughout the neuronal plasma membrane including the excitatory post synapse. Primary hippocampal neurons at DIV 14–20 were transduced with CRHR1-WT and CRHR1-STAVA using AAV8. (A) The spatial pattern of CRHR1-WT and CRHR1-STAVA expression visualized by immunostaining of the GFP tag was confirmed using an anti-CRHR1 antibody. (B) Microtubule-associated protein 2 (MAP2) staining revealed the dendritic presence of the receptor. (C) Co-staining with the axon initial segment marker ankyrin G demonstrated CRHR1-WT and CRHR1-STAVA localization within axons. (D) The presynaptic marker synapsin indicated that CRHR1-WT and CRHR1-STAVA are present in the adjacent post synapse but not in the presynaptic axon terminal. (E) CRHR1-WT and CRHR1-STAVA did not co-localize with the inhibitory postsynaptic marker gephyrin but (F) they co-localized with the MAGUK and excitatory postsynaptic marker PSD95 in spines. (G) CRHR1-WT and mutant co-localized with the candidate interaction partner SAP97.

Using heterologous expression in HEK293 cells we confirmed the PDZ binding motif-dependent interaction of CRHR1 with PSD95, SAP97, SAP102, PSD93, and MAGI2. We next investigated whether CRHR1 is co-localized with the interaction partners on the subcellular level and whether this depends on the PDZ binding motif. Therefore, we used primary hippocampal neurons, which endogenously express CRHR1, and identified interactors (Fig 2). The reliable detection of endogenous CRHR1 expression at the protein level was not possible due the low expression and a lack of specific and sufficiently sensitive antibodies [5]. Therefore, we took advantage of adeno-associated viruses (AAVs) to express GFP-tagged CRHR1-WT in cultured hippocampal neurons. To express CRHR1 in neurons that endogenously express the receptor, i.e., glutamatergic neurons, we prepared primary neurons from Nex-Cre mice which express Cre recombinase in glutamatergic neurons only. Transduction of these primary neurons with Cre-dependent AAVs, which are based on the DIO (double-floxed inverse open reading frame) expression system, restricted the expression of CRHR1 to glutamatergic neurons. CRHR1 was subsequently detected using an antibody directed against the GFP tag. In parallel, a CRHR1-specific antibody was used that was sufficiently sensitive to detect overexpressed exogenous CRHR1. This antibody fully recapitulated the localization pattern revealed by the GFP antibody. CRHR1 expression was detected in the plasma membrane throughout the neuron, including dendrites, axons, and cell body (Fig 7A–7E). CRHR1 was also present in the postsynaptic densities of mature spines, where it co-localized with PSD95 and SAP97 (Fig 7F and 7G). In addition, no CRHR1 expression was detected in inhibitory synapses, as indicated by the lack of co-staining with gephyrin (Fig 7E). To investigate whether the PDZ binding motif is necessary for the correct subcellular localization, we transduced primary hippocampal neurons with another AAV to express the GFP-tagged CRHR1-STAVA mutant. However, the subcellular distribution of CRHR1-STAVA was indistinguishable from the localization of WT CRHR1 (Fig 7H–7N), suggesting that the PDZ binding motif does not control the gross localization of CRHR1 in primary neurons under basal conditions.

### PDZ binding motif is essential for clustering of CRHR1 with interaction partners

**Fig 8:**
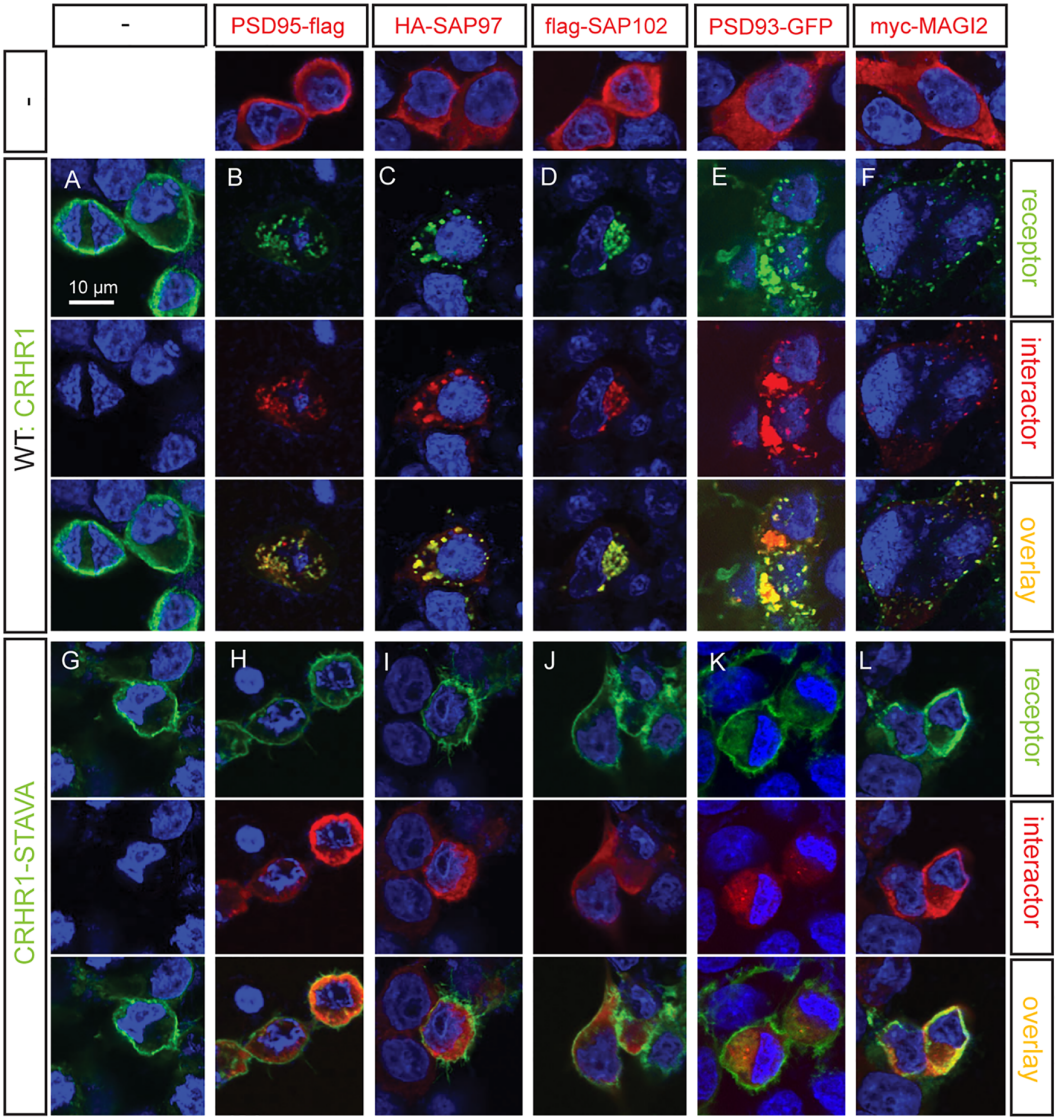
CRHR1 PDZ binding motif is required for clustering with interacting MAGUKs. CRHR1-WT (A), CRHR1-STAVA (G), or the interacting proteins (first row) were transiently transfected alone. (B–F) Wild-type CRHR1 or (H–L) CRHR1-STAVA were transiently transfected in HEK293 together with (B, H) PSD95-flag, (C, I) HA-SAP97, (D, J) flag-SAP102, (E, K) PSD93-GFP and (F, L) myc-MAGI2. CRHR1-WT co-transfection with the respective interacting partner resulted in a co-clustering of both proteins (B–F). (H–L) Co-transfection of CRHR1-STAVA with the respective interacting partner did not result in any clustering, but both proteins appeared with a subcellular distribution similar to the individual transfections. Immunostaining was performed against the HA tag of HA-CRHR1 or HA-CRHR1-STAVA when co-transfected with PSD95-flag or flag-SAP102. Immunostaining was performed against the flag tag of flag-CRHR1 or flag-CRHR1-STAVA when co-transfected with PSD93-GFP, HA-SAP97, or myc-MAGI2 respectively.

MAGUKs have been demonstrated to play a role in the clustering of other GPCRs such as the serotonin (5-hydroxytryptamine) receptor 2A (5-HT2A) [29]. To test whether this is also the case for CRHR1, we performed a clustering assay. CRHR1-WT and the CRHR1-STAVA mutant localized to the plasma membrane when expressed alone in HEK293 cells (Fig 8A and 8G). Furthermore, PSD95, SAP97, SAP102, PSD93 and MAGI2 exhibited a mainly cytosolic localization pattern (Fig 8, first row). However, when CRHR1 was co-expressed with individual MAGUKs, both CRHR1 and the interactor were redistributed and localized in clusters intracellularly and partly also at the membrane (Fig 8B–8F). In contrast, when CRHR1-STAVA was co-expressed with PSD95, SAP97, SAP102, PSD93, or MAGI2, there was neither a change in the distribution of CRHR1 nor of the interacting MAGUKs. All proteins were located in a similar manner, as if they were expressed alone (Fig 8H–8L). This clustering pattern depending on an intact PDZ binding motif demonstrated the functional relevance of the PDZ-mediated interaction between CRHR1 and the identified MAGUKs.

## Discussion

In this study, we found new interacting partners of the CRHR1, i.e., MAGUKs PSD95, SAP97, SAP102, PSD93 and MAGI2. These scaffold proteins are co-expressed with CRHR1 in excitatory and inhibitory neurons of the adult mouse brain as well as in primary hippocampal neurons. In the latter, CRHR1 is found throughout the plasma membrane, including the excitatory post synapse, where the receptor co-localizes with MAGUKs. The interaction of MAGUKs with the functional C-terminal class I PDZ binding motif of CRHR1 via different PDZ domains induced their co-clustering with CRHR1. These results provide strong evidence that the *in vivo* interaction of CRHR1 with the identified MAGUKs is highly likely and thus presumably involved in modulating CRHR1 function.

MAGUKs are crucial for the assembly of core signaling complexes and their disturbance has been implicated in synaptopathies, which cause major psychiatric, neurological and childhood developmental disorders [30]. Altered expression in psychiatric illness has been similarly reported for several MAGUKs [31]. Hence, the physical interaction of CRHR1 with multiple MAGUKs suggests that some of the emotional disturbances observed across mental disorders could be linked to impaired CRHR1 function.

Individual MAGUKs have been shown to specifically interact with distinct proteins via specific PDZ domains. For example, SAP97 is the only MAGUK that can directly bind to the PDZ binding motif A-T-G-L of the AMPA receptor subunit GluA1 (GluR1) [32]. For this interaction, the PDZ2 domain and to a lesser extent also the PDZ1 domain is important [33]. In our study, SAP97 interacted via the PDZ2, but not the PDZ1 domain with the PDZ binding motif of CRHR1. During this study, the PDZ binding motif-dependent interaction of CRHR1 with full-length SAP97 was also demonstrated by Dunn and colleagues [34]. They observed that the CRHR1 interaction had a direct impact on CRHR1 endocytosis but not on cAMP production. Interestingly, SAP97 also influenced CRHR1 downstream ERK1/2 signaling, albeit independently of the C-terminal PDZ binding motif [34].

Regarding PSD95, the N-terminal tandem PDZ domains 1 and 2 are necessary and sufficient for interaction with CRHR1. PSD95 generally interacts with other proteins mainly via these N-terminal tandem PDZ domains, e.g. with the C-terminal class I PDZ binding motifs S-S-A-V of the G-protein-coupled receptor 30 (GPR30) [35] and I-S-T-L of the somatostatin receptor 1 (SSTR1) [26] which are different from the CRHR1´s motif S-T-A-V but also classical class I PDZ binding motifs. The third PDZ domain comprises additional properties that determine its distinct ligand specificity [36, 37]. PSD95 similarly binds via its first and second PDZ domain to the C-terminal class I PDZ binding motif E-S-D-V of NMDA receptor subtype 2A (GluN2A/NR2A) and GluN2B. In particular, the first eight amino acids of PDZ2 have been revealed to be of major importance for this interaction by affecting the folding of the PDZ domain [38, 39]. Our recent characterization of the three PDZ domains of PSD95 with regard to binding kinetics and affinity toward C-terminal domains of numerous GPCRs further underscored the importance of the first two PDZ domains [26]. However, the prediction of PDZ domain interactions is complicated by the fact that amino acids upstream of the well-established PDZ binding motifs are also relevant for the interaction [37, 40].

Amongst others, PDZ-mediated interactions are regulated by phosphorylation of the PDZ binding motif [41]. The interaction of PSD95 with stargazin can be disrupted via a phospho-mimicking substitution Thr312Glu within the PDZ binding motif T-P-V [42]. In our study the mimicking of phosphorylation at Thr413 but not at Ser412 disrupted the interaction of CRHR1 with PSD95 indicating that phosphorylation might regulate the interaction of CRHR1 with MAGUKs. However, it has to be considered that this disruption might just be the consequence of altering the amino acid at position -2 of the class I PDZ binding motif. In the case of the CRHR1 substitution of Thr413 by a phosphorylation preventing alanine also disrupted the interaction what is often not tested in phospho-mimicking studies [42]. Similarly, another study demonstrated by using a phosphorylation site-specific antibody that the negatively regulated interaction of PSD95 with GluN2B depends on phosphorylation within the PDZ binding motif S-D-V [43] and the phosphorylation of the amino acid at position -2 of the PDZ binding motif often inhibited PDZ-mediated interactions [41]. Previous studies demonstrated that PSD95 inhibits the agonist-induced 5-HT2AR internalization via PDZ domain interactions, thus modulating the localization of the receptor [29]. The PDZ binding motif of the 5-HT2A receptor has also been demonstrated to be essential for dendritic targeting in cortical pyramidal neurons [44].

In contrast to 5-HT2AR, CRHR1 was present throughout the neuronal plasma membrane in axons and dendrites, including the excitatory post synapse, independent of its intact PDZ binding motif. We could demonstrate that CRHR1 is co-localized with the interacting partners PSD95 and SAP97 within dendritic spines. This is in line with previous studies demonstrating the co-localization of CRHR1 with PSD95 on dendritic spine heads of hippocampal CA3 neurons [45, 46]. However, in our case, CRHR1 expression was not restricted to spine heads but it extended to the dendritic shaft. Nevertheless, this result has to be taken with some caution, as it could be related to AAV-mediated overexpression in primary hippocampal neurons, which was indispensable to visualize CRHR1 localization because of low endogenous CRHR1 expression levels and the unavailability of reliable antibodies [5]. In addition to PSD95 and SAP97, we identified and characterized SAP102, PSD93, and MAGI2 as novel previously unknown interactors of CRHR1. These MAGUKs have also been shown to interact with numerous transmembrane receptors containing class I PDZ binding motifs, including NMDA receptors subunits GluN2A, GluN2B [36, 47], and GluN2C [48].

CRHR1 localization in the excitatory postsynaptic density is important for its interaction with MAGUKs as this is the major site where they act as central scaffolds for receptors, ion channels, and signaling proteins. The capability of MAGUKs to cluster different proteins is a property that is also observed in heterologous expression systems. PSD95 and PSD93 have been accordingly demonstrated to induce cell surface clustering with class I PDZ binding motif containing GluN2B in COS-7 cells [47]. Although the PDZ binding motif was not affecting CRHR1 localization in primary neurons, CRHR1 clustering with the identified MAGUKs was detected in HEK293 cells. The clustering was completely disrupted when the CRHR1-STAVA mutant was used, emphasizing the importance of the C-terminal PDZ binding motif. Therefore, we hypothesize that MAGUKs can anchor CRHR1 to larger signaling complexes, positioning the receptor in close vicinity to other receptors and ion channels and linking it to the intracellular signaling machinery.

The recently described impact of CRHR1 on spine dynamics suggests the involvement of interactions with MAGUKs. Stress or direct CRH treatment induce spine loss, an effect that is abolished by blocking CRHR1. These structural changes are causally involved in the stress-induced impairment of synaptic plasticity and memory deficits. The loss of dendritic spines involves destabilization of F-actin which is triggered by CRH-mediated activation of the small GTPase RhoA after CRH treatment [49, 50]. In addition, this process requires network activity and the CRH-mediated activation of NMDA receptors, which in turn activates the calpain-mediated breakdown of spine actin-interacting proteins such as spectrin. As every identified MAGUK interacts with NMDA receptors [28, 36, 38, 47, 51–53], it is likely that the functional connection of CRHR1 to NMDA receptors in conjunction with stress-induced spine loss is conveyed by MAGUKs [54]. MAGUKs are linked to the cytoskeleton and actin through proteins that bind to their SH3 and GuK domains such as the guanylate kinase-associated protein (GKAP) or the spine-associated RapGAP (SPAR) [55–57]. Many signaling molecules are clearly involved in the CRH–CRHR1-mediated spine loss, corresponding to the fact that different temporal and spatial signal compositions determine divergent signaling pathways. The described mechanisms are either calcium-dependent or -independent and probably account for the selection of stress effects resulting in spine loss.

Today it is widely accepted that GPCRs can form homo- or heterodimers or even higher-order oligomers. Dimerization can alter ligand binding and the interaction with different effector proteins including G proteins or β-arrestins [58]. The interaction of GPCRs via binding to MAGUKs offers an intriguing alternative pathway for cooperation between different GPCRs. Along these lines, Magalhaes and colleagues illustrated that CRHR1 regulates anxiety-related behavior sensitizing 5HT2R signalling, which required intact PDZ binding motifs of both receptors [59]. SAP97 has been recently excluded as the particular MAGUK responsible for this effect [60]. However, other MAGUKs which we identified as novel interactors of CRHR1 have also been shown to interact with 5-HT2Rs including PSD95, SAP102 and MAGI2 [29, 61]. In a similar manner CRHR1 has been demonstrated to interact via PSD95 with the GPR30 [35]. It is highly likely that CRHR1 interacts via its PDZ binding motif in a tripartite complex with other GPCRs such as 5-HT2Rs or SSTR1, which we found to harbor a PDZ binding motif that interacts with PSD95 and which is located in dendritic spines [26, 59].

The mechanism regarding the coupling of a specific G protein to CRHR1 and subsequent signaling via the PLC-PKC or adenylyl cyclase-PKA cascade [7] remains unclear, but it is known that several MAGUKs, including PSD95 and SAP97, can interact with A-kinase anchor proteins (AKAPs) via their SH3 and GuK domains [62]. AKAP79/150 appears to function as a scaffold protein for PKC and PKA [63, 64]. Therefore, it is intriguing to speculate that CRHR1 signaling is modulated by specific MAGUKs that bring particular signaling molecules into close proximity with CRHR1. These signaling microdomains may reflect another level of subcellular compartmentalization.

Altogether, we established multiple MAGUKs as CRHR1 interaction partners, unraveled their key PDZ domains relevant for the interaction, and ultimately validated the C-terminal PDZ binding motif as a central module of the receptor to interact with the intracellular signaling machinery. CRHR1 co-localization with different MAGUKs within the brain hints toward the physiological relevance of the identified interactions. The future challenge will be to understand the specificity of CRHR1–MAGUK interactions and composition of related signaling complexes and the mechanism by which they affect CRHR1 signaling. Moreover, it will be of major interest to investigate the mechanism by which these interactions modulate anxiety-related behavior. It is intriguing to hypothesize that differences in interactions with MAGUKs are shaping previously described CRHR1-dependent anxiogenic or anxiolytic circuits [5]. These findings will help to unravel the role of CRHR1 in stress-related circuits on the molecular level, a prerequisite for understanding its role in stress-related pathologies.
